# Trace Amine-Associated Receptor 1 Trafficking to Cilia of Thyroid Epithelial Cells

**DOI:** 10.3390/cells10061518

**Published:** 2021-06-16

**Authors:** Maria Qatato, Vaishnavi Venugopalan, Alaa Al-Hashimi, Maren Rehders, Aaron D. Valentine, Zeynep Hein, Uillred Dallto, Sebastian Springer, Klaudia Brix

**Affiliations:** Department of Life Sciences and Chemistry, Focus Area HEALTH, Jacobs University Bremen, Campus Ring 1, D-28759 Bremen, Germany; m.qatato@dkfz-heidelberg.de (M.Q.); v.venugopalan@jacobs-university.de (V.V.); a.alhashimi@jacobs-university.de (A.A.-H.); m.rehders@jacobs-university.de (M.R.); a.valentine@jacobs-university.de (A.D.V.); z.hein@jacobs-university.de (Z.H.); willred.com@hotmail.com (U.D.); s.springer@jacobs-university.de (S.S.)

**Keywords:** cilia, G protein-coupled receptors, green fluorescent protein, thyroid auto-regulation, thyroid epithelial cells, trace amine-associated receptor 1, trafficking

## Abstract

Trace amine-associated receptor 1 (rodent Taar1/human TAAR1) is a G protein-coupled receptor that is mainly recognized for its functions in neuromodulation. Previous in vitro studies suggested that Taar1 may signal from intracellular compartments. However, we have shown Taar1 to localize apically and on ciliary extensions in rodent thyrocytes, suggesting that at least in the thyroid, Taar1 may signal from the cilia at the apical plasma membrane domain of thyrocytes in situ, where it is exposed to the content of the follicle lumen containing putative Taar1 ligands. This study was designed to explore mouse Taar1 (mTaar1) trafficking, heterologously expressed in human and rat thyroid cell lines in order to establish an in vitro system in which Taar1 signaling from the cell surface can be studied in future. The results showed that chimeric mTaar1-EGFP traffics to the apical cell surface and localizes particularly to spherical structures of polarized thyroid cells, procilia, and primary cilia upon serum-starvation. Moreover, mTaar1-EGFP appears to form high molecular mass forms, possibly homodimers and tetramers, in stably expressing human thyroid cell lines. However, only monomeric mTaar1-EGFP was cell surface biotinylated in polarized human thyrocytes. In polarized rat thyrocytes, mTaar1-EGFP is retained in the endoplasmic reticulum, while cilia were reached by mTaar1-EGFP transiently co-expressed in combination with an HA-tagged construct of the related mTaar5. We conclude that Taar1 trafficking to cilia depends on their integrity. The results further suggest that an in vitro cell model was established that recapitulates Taar1 trafficking in thyrocytes in situ, in principle, and will enable studying Taar1 signaling in future, thus extending our general understanding of its potential significance for thyroid autoregulation.

## 1. Introduction

Cilia of thyroid epithelial cells are involved in the regulation and maintenance of thyroid homeostasis and intact follicle structure [1,2,3,4,5,6]. Thyrocytes in well-polarized states expose one primary immotile cilium per cell that is identified by the cilia marker acetylated alpha-tubulin [1,2,4]. The primary cilium extends from the apical surface of polarized thyroid epithelial cells, e.g., in confluent cultures of Fisher rat thyroid (FRT) cells. Upon long-term FRT cell culture, follicle-like structures (FLS) are formed whereby thyrocytes build a monolayer around an extracellular lumen into which cilia extend [1]. Hence, cilia of cultured thyrocytes in vitro mimic the in situ-localization of primary cilia at the apical surface of thyrocytes in the sphere-like follicles of thyroid tissue [1,2,4].

Alterations of cilia or changes in their frequency are indicative of thyroid diseases, ranging from dysfunctional thyroid states to neoplastic pathologies [2,7]. While such correlations of thyroid pathologies with altered cilia length and numbers are important as diagnostic criteria in thyroid disease, little is known about the molecular mechanisms that connect cilia with altered thyroid states. To this end, we proposed a thyroid auto-regulatory mechanism that encompasses cilia as sensory extensions of thyrocytes probing the molecular state of the thyroid hormone (TH) precursor protein, thyroglobulin, which is stored in the thyroid follicle lumen [1,4,5]. The trace amine-associated receptor 1 (TAAR1 in human, mTaar1 in mouse, rTaar1 in rat), a G protein-coupled receptor (GPCR), has been suggested as the ciliary molecule that senses the state of luminal thyroglobulin, thereby enabling thyroid function by initiating or terminating its proteolytic utilization for TH liberation [1,5]. It is of note that mTaar1 and the basolateral GPCR thyroid-stimulating hormone (TSH) receptor co-regulate thyroid function in vivo [8].

TAAR1 has been identified to be susceptible to activation by a variety of biogenic amines [9,10,11,12]. Attempts to understand the physiological role of TAAR1, its trafficking and subcellular localization have been challenged by the protein’s weak cell surface expression in vitro [13], and the difficulty in achieving stable TAAR1 expression in heterologous systems [11]. Nonetheless, the limited in vitro studies available assume that TAAR1/Taar1 retains an intracellular localization. The exact transport pathways, however, as well as the main subcellular TAAR1 localization along the secretory route, and whether dimer or oligomer formation, either with itself or other GPCRs, is required for productive transport to the cell surface remain an important field for investigations.

We have previously shown that, at steady state, Taar1 localizes to compartments of the secretory pathway and, prominently, to the cilia of mouse and rat thyrocytes [1,4]. Using rat thyroid epithelial cell lines, we further showed that cell surface expression of rTaar1 in vitro depends on intact cilia, reminiscent of Taar1′s in situ localization in rodent thyroid tissue [1,4]. Since previous studies by us and others are further suggestive of an essential role of cilia in thyroid function regulation in man, mouse and rat, it is particularly important to better understand TAAR1/Taar1 trafficking to the cilia of thyroid epithelial cells [2,4,5,7,8].

The present study was designed to test the proposal of mTaar1 being transported along the secretory pathway in a heterologous system of stable *mTaar1* expression. To this end, a construct coding for mTaar1 tagged with enhanced green fluorescent protein (EGFP) on its C-terminus was used to stably express *mTaar1-EGFP* in normal thyroid epithelial (Nthy-ori 3-1) and papillary thyroid carcinoma (KTC-1) cell lines, bearing characteristics of non- and well-polarized thyroid epithelial cells, respectively [14]. The subcellular localization of mTaar1-EGFP and its transport pathways were investigated in these cell lines at steady state and in pulse-chase experiments. The results show that mTaar1-EGFP reaches spherical structures at the apical plasma membrane of thyrocytes, referred to as procilia. When ciliogenesis was promoted by serum-starvation, mTaar1-EGFP was transported to elongated structures, co-stainable with the cilia markers acetylated α-tubulin or ARL13B, in both *mTaar1-EGFP* stably expressing cell lines. Thus, the presence of cilia in KTC-Z, the stably *mTaar1-EGFP* expressing and well-polarized human thyrocytes, promotes mTaar1-EGFP trafficking to this specific cell surface localization and maintains it at the cellular appendages. These results corroborate our previous findings with mouse and rat thyrocytes in situ and in vitro.

However, transient *mTaar1-EGFP* expression in cilia-bearing FRT cells results in endoplasmic reticulum (ER) retention, thereby hindering mTaar1-EGFP’s transport to the apical plasma membrane. Hence, we further aimed at delineating possible co-trafficking partners of the same GPCR family in thyrocytes. Taar proteins have been classified into three phylogenetic subgroups [15]. Consequently, Taar5 and Taar8b were picked as representatives of the two other phylogenetic subgroups, besides Taar1. Contrary to Taar1, both Taar5 and Taar8b were previously shown to reach the cell surface in transiently expressing HEK 293T cells [16,17]. Interestingly, co-expression of *mTaar1-EGFP* and the related hemagglutinin (HA)-tagged mTaar5, *HA-mTaar5*, promotes Taar1’s ability to reach cilia in transiently co-expressing polarized FRT cells. Therefore, we propose that oligomerization of mTaar1 occurs early in the secretory pathway and promotes Taar1 trafficking to cilia of thyroid epithelial cells.

## 2. Materials and Methods

All studies were performed in the S1 and S2 laboratories of Jacobs University Bremen as registered with the Authorities of the City State of Bremen (Senatorin für Gesundheit, Frauen und Verbraucherschutz der Hansestadt Bremen, Bremen, Germany) under registration numbers 513-30-00/2-15.32 and 517/2-15.43 to K.Br. and S.Sp., respectively, as the responsible project leaders.

### 2.1. Vector Construction

Plasmids coding for mouse Taar1, Taar5 or Taar8b, with an HA-tag fused to the N-terminus, cloned into a pcDps expression vector (*p*HA-mTaar1, *p*HA-mTaar5 and *p*HA-mTaar8b) were described previously [16,17].

The *p*HA-mTaar1 plasmid was employed as a template to amplify the mouse *Taar1*, *Taar5* or *Taar8b* cDNA sequence, omitting the stop codon, while providing overhangs complementary to the *XhoI* and *BamHI* restriction sequences to enable ligation into the pEGFP-N1 (Clontech, Heidelberg, Germany) expression vector using T4 DNA ligase (EL0011, Thermo Scientific, Schwerte, Germany). The resultant plasmid coded for a chimeric protein with full-length mouse Taar1, covalently linked to the EGFP tag by a 12-amino acid long spacer peptide linker (*p*mTaar1-EGFP). The sequence was confirmed using standard pEGFP-N1 forward and reverse primers at Eurofins Genomics (Ebersberg, Germany).

Similarly, the sequence coding for full-length mTaar1 minus the stop codon was cloned into a modified puc2CL6Ipwo lentiviral vector [18,19] at *XhoI* and *AgeI* sites of insertion (5′-end and 3′-end, respectively), to obtain a construct coding for the chimeric protein consisting of full-length mTaar1, covalently linked to EGFP by a 12-amino acid long spacer peptide linker (mTaar1-EGFP in puc2CL6Ipwo). Sequences were confirmed at Eurofins Genomics (Ebersberg, Germany) using oSF031Fwd (5′-CGGCGCGCCAGTCCTCCG) and oSF031Rev (5′-TAGACAAACGCACACCGG) sequencing primers.

### 2.2. Cell Culture

Fisher rat thyroid (FRT) cells were grown in F-12 Coon’s media (F-6636, Sigma-Aldrich, Steinheim, Germany), supplemented with 31.9 mM NaHCO_3_, 5% Fetal Bovine Serum (FBS; F7524, Sigma-Aldrich, Steinheim, Germany), 10 μg/mL insulin (I6634, Sigma-Aldrich, Steinheim, Germany), 5 μg/mL transferrin (11107-018, Invitrogen, Darmstadt, Germany), 10 ng/mL somatostatin (S1763, Sigma-Aldrich), 10 ng/mL glycyl-histidyl-lysine (G7387, Sigma-Aldrich, Steinheim, Germany), and 10 nM hydrocortisone (H-0135, Sigma-Aldrich, Steinheim, Germany).

HEK 293T cells (ACC 635, DSMZ, Braunschweig, Germany), were cultured in Dulbecco’s modified Eagle’s medium (DMEM; BE12-604F, Lonza, Verviers, Belgium), supplemented with 2 mM glutamine and 10% FBS.

KTC-1 [20,21] and Nthy-ori 3-1 [22] cells were cultured in RPMI 1640 medium with 2 mM L-glutamine (BE12-702F, Lonza, Verviers, Belgium), supplemented with 10% FBS, 100 U/mL penicillin and 0.1 mg/mL streptomycin (P0781, Sigma-Aldrich, Steinheim, Germany).

All cell lines were incubated at 37 °C and 5% CO_2_ in a moisturized atmosphere, unless otherwise indicated.

For trafficking studies, cell lines were grown on sterile coverslips until confluent, then incubated overnight at 18 °C in Gibco’s CO_2_-independent culture medium (18045, Thermo Fisher Scientific, Schwerte, Germany), supplemented with 10% FBS and 1 µg/mL puromycin, and shifted to 37 °C subsequently for the indicated time periods. Cells were fixed in 4% paraformaldehyde (PFA) in 200 mM HEPES, pH 7.4, at t = 0 min, 15 min, 30 min, 45 min, 1.0 h, 1.5 h, 2.0 h, 3.0 h and 4.0 h, respectively, post-temperature shift, and immunolabeled with compartment-specific markers, as described below.

For experiments on cilia markers, cell lines were serum-starved for 48 h in order to promote ciliogenesis before fixation in 4% PFA for 20 min at room temperature and in ice-cold methanol for 5 min at −20 °C, and immunolabeling, as described below.

### 2.3. Transient Transfection by Electroporation

Cells were incubated in 1 mL 0.25% trypsin in EDTA solution (T4549, Sigma-Aldrich, Steinheim, Germany) at 37 °C until they detached. They were washed in calcium- and magnesium-free PBS (CMF-PBS) consisting of 0.15 M NaCl, 2.7 mM KCl, 1.5 mM NaH_2_PO_4_, 8.1 mM Na_2_HPO_4_, pH 7.4, then 1 × 10^6^ cells were resuspended in 150 μL cytomix solution (120 mM KCl, 10 mM KH_2_PO_4_, 5 mM MgCl_2_, 25 mM HEPES, 2 mM EGTA, 2 mM ATP; A-2383, Sigma-Aldrich, Steinheim, Germany), and 5 mM oxidized glutathione (G4626, Sigma-Aldrich, Steinheim, Germany, pH 7.2) [23] with 10 μg DNA. Resuspended FRT cells were pulsed twice at 700 V for 200 μs using a Multiporator^®^ (940000505, Eppendorf, Hamburg, Germany) using electroporation cuvettes with 2 mm gap width and 400 µL capacity (940001013, Eppendorf, Hamburg, Germany). Following 10 min recovery on ice, cells were seeded onto sterile coverslips in 6-well plates, at an approximate density of 5 × 10^5^ cells per 2 mL media per well. Cells were fixed as described above 48 h post-transfection.

### 2.4. Lentiviral Transduction

KTC-1 and Nthy-ori 3-1 cells were transduced as previously described [14,18,19,24]. In brief, HEK 293T cells were transfected with 6 µg of each of the following plasmids: puc2CL6IP-mTaar1-EGFP, pCDNL-BH and vesicular stomatitis virus G, with 45 µL of a 1 mg/mL polyethylenimine, branched solution (PEI; 408,727 Sigma-Aldrich, Steinheim, Germany), as transfection reagent. The virion-rich supernatant of HEK 293T cells was collected 48 h post-transfection and filtered through a 0.45 µm filter before being applied to 70% confluent KTC-1 and Nthy-ori 3-1 cells. Transduced cells underwent selection with 1 µg/mL puromycin (0240.2, Carl Roth, Karlsruhe, Germany) in culture medium.

Henceforth, the acronyms KTC-Z and Nthy-Z will be used when referring to transduced, *mTaar1-eGFP*-expressing KTC-1 and Nthy-ori 3-1 cells, respectively. KTC-Z and Nthy-Z cells were cultured in RPMI 1640 medium (Lonza, Verviers, Belgium) supplemented with 10% FBS, in the presence of penicillin and streptomycin. When cells were thawed from frozen stocks, transduction efficacy was controlled by FACS and cells were eventually re-selected using complete culture medium supplied with 1 µg/mL puromycin.

### 2.5. Cytochemistry and Indirect Immunofluorescence

Following fixation, cells were washed 3 × 5 min by incubation with CMF-PBS and blocked in 3% bovine serum albumin (BSA; 3854, Carl Roth, Karlsruhe, Germany) in CMF-PBS for 60 min at 37 °C.

Cells grown on coverslips were incubated with primary antibodies diluted in 0.1% BSA in CMF-PBS overnight at 4 °C. For compartment-specific immunolabeling, mouse anti-human GM130 (1:100; 610822, BD Transduction Laboratories, Allschwil, Switzerland), mouse anti-acetylated α-tubulin (1:100; T7451, clone 6-11 B-1, purified from hybridoma cell culture, Sigma-Aldrich, Steinheim, Germany), and mouse anti-human LAMP-2 (1:100; H4B4, DSHB—Developmental Studies Hybridoma Bank, University of Iowa, Iowa City, IA, USA) antibodies were used. In order to label cell surface glycoproteins, cells were treated with 10 µg/mL of biotin-conjugated Concanavalin A (ConA) from *Canavalia ensiformis* (C2272, Sigma-Aldrich, Steinheim, Germany) for 30 min at 4 °C. In order to visualize ciliary extensions, cells were stained with mouse anti-acetylated α-tubulin (1:100; T7451, Sigma-Aldrich, Steinheim, Germany), rabbit anti-CP110 (1:50; 12780-1-AP, Proteintech through Thermo Fisher Scientific, Bremen, Germany), and rabbit anti-ARL13B (1:50; 17711-1-AP, Proteintech through Thermo Fisher Scientific, Bremen, Germany).

After washing with 0.1% BSA in CMF-PBS, cells were incubated with Alexa 488- or Alexa 546-conjugated secondary antibodies (1:250; Molecular Probes, Karlsruhe, Germany), or with Alexa Fluor 546-conjugated streptavidin (1:200; S-11225, Molecular Probes, Karlsruhe, Germany) for ConA label detection, for 1 h at 37 °C together with 5 μM Draq5™ (Biostatus Limited, Shepshed, UK) to counter-stain nuclear DNA. Primary antibodies were omitted in negative controls.

The cells on coverslips were mounted with embedding medium consisting of 33% glycerol, 14% Mowiol in 200 mM Tris-HCl, pH 8.5 (Hoechst AG, Frankfurt, Germany). The slides were analyzed by confocal laser scanning microscopy using Argon and Helium-Neon, or diode lasers (LSM 510 Meta; Carl Zeiss Jena GmbH, Jena, Germany; LSM 980 with Airyscan 2 and Multiplex; Carl Zeiss Microscopy GmbH, Oberkochen, Germany). Images were obtained at a pinhole setting of 1 Airy unit and at a resolution of 1024 × 1024 pixels or using high-resolution Airyscan modes. Micrographs were analyzed with the LSM 510 software, release 3.2 (Carl Zeiss Jena GmbH, Jena, Germany) and with the LSM 980 ZEN 3.2 software (Carl Zeiss Microscopy GmbH, Oberkochen, Germany).

### 2.6. Cell Lysate Preparation, SDS-PAGE and Immunoblotting

Following washing in ice-cold PBS, cells were scraped off the 10 cm Petri dishes and collected in 500 µL lysis buffer, consisting of 50 mM Tris (pH 6.8) with 0.2% Triton-X 100 (TX-100) and supplemented with protease inhibitors (0.2 μg/mL aprotinin, 10 μM E-64 and 1 μM pepstatin A and 2 mM EDTA). The cell lysates were incubated for 1 h at 4 °C with constant rotation, and cleared by centrifugation for 10 min at 10,000× *g* at 4 °C. The supernatants were collected and protein content was determined according to the Neuhoff assay [25].

Protein samples were prepared in Laemmli sample buffer [26] (10 mM Tris-HCl (pH 7.6), 0.5% sodium dodecyl sulphate (SDS), 25 mM dithiotreitol (DTT), 10% glycerol, 25 mg/mL bromophenol blue) and heated for 5 min at 95 °C prior to loading onto 12.5% SDS-polyacrylamide gels, which were then semi-dry blotted onto nitrocellulose membranes [27]. Equal loading of the lanes and successful protein transfer were assessed by staining of the membranes with Ponceau S solution for 10 min at room temperature (A2395, AppliChem, Darmstadt, Germany). Pre-stained protein standards covering a broad range of 11–245 kDa (P7712S, New England Biolabs GmbH, Frankfurt, Germany) were used as molecular mass markers. Non-specific binding sites were blocked in 5% blotting grade milk powder (T1452, Carl Roth, Karlsruhe, Germany) in PBS-special buffer (pH 7.5), CMF-PBS containing 0.3% Tween-20 solution (9127.2, Carl Roth, Karlsruhe, Germany) (PBS-T) overnight at 4 °C, except for samples subjected to biotinylation, which were blocked in a 3% BSA in PBS-T solution instead. Membranes were incubated in primary antibodies, i.e., rabbit anti-GFP (1:1000; ab209, Abcam, Cambridge, UK) or mouse anti-GFP (1:1000; 1814460, Roche Diagnostics GmbH, Mannheim, Germany) diluted in PBS-T were used overnight at 4 °C, washed in PBS-T buffer 6 × 5 min on a shaker at room temperature, then incubated with goat anti-rabbit or goat anti-mouse (respectively), horseradish (HRP)-conjugated IgG secondary antibody (1:5000; Southern Biotech, Birmingham, USA) for one hour while constantly rotating at room temperature. Alternatively, the membranes were incubated in HRP-conjugated streptavidin (1:10,000; S-5512, Sigma-Aldrich, Steinheim, Germany) in PBS-T. Following the washing steps, membranes were incubated with SuperSignal™ West Pico PLUS Chemiluminescent Substrate (34580, Thermo Scientific, Rockford, IL, USA) for 2 min at room temperature, and scanned using C-DiGit Blot Scanner from Li-COR Biosciences and the Image Studio Lite software version 5.2 (Lincoln, NE, USA).

### 2.7. Cell Surface Biotinylation and Streptavidin Pull-Down Experiments

Cell surface biotinylation was performed according to a modified protocol described elsewhere [28]. In brief, KTC-1, KTC-Z, Nthy-ori 3-1 and Nthy-Z cells were cultured in biotin-free medium (DMEM supplemented with 10% FBS and 1 µg/mL puromycin for transduced “Z” cells) continuously for 14 days prior to commencing the experiments. Cells were grown in 10 cm Petri dishes until ~70%–90% confluent. The cells were then washed in cold PBS 2 × 30 min and incubated with 200 µg/mL biotinamidohexanoic acid 3-sulfo-N-hydroxysuccinimide ester sodium salt (B1022, Sigma-Aldrich, Steinheim, Germany) in PBS for 1 h at 4 °C with gentle shaking. Non-biotinylated controls were incubated in parallel in PBS only. Then, cells were briefly rinsed in PBS, and washed with 10 mM L-lysine (L5501, Sigma-Aldrich, Steinheim, Germany) in PBS solution 4 × 10 min to quench unbound biotin. Finally, the cells were incubated in lysis buffer (50 mM Tris, pH 6.8, with 0.2% TX-100, containing protease inhibitors as specified above), and collected in 2 mL microcentrifuge tubes to complete cell lysis and protein extraction, as described above. Cell lysates were subsequently used for SDS-PAGE and immunoblotting (see above).

For streptavidin pull-down, cells were homogenized in cold homogenization buffer (250 mM sucrose, 20 mM HEPES, 1 mM EDTA, pH 7.4), supplemented with protease inhibitors as specified above, using a hand-held homogenizer at 500 rpm for 2 × 30 s, on ice. Homogenates were cleared by centrifugation for 15 min at 10,000× *g* at 4 °C. The supernatant was collected and protein content was determined according to the Neuhoff assay [25].

Streptavidin-precipitation was carried out using the µMACS streptavidin kit (130-074-101; Milteny Biotech, Bergisch-Gladbach, Germany) according to the manufacturer’s protocol. Cold µMACS streptavidin MicroBeads solution was added to the cell homogenates in a ratio of 1:3 on ice and mixed by slowly pipetting up and down. The µ-column was placed in the magnetic field of the μMACS separator and prepared by rinsing it with 100 µL equilibration buffer prior to protein application, followed by two rinsing steps with 100 µL homogenization buffer (without proteinase inhibitors). The magnetically labeled complexes, i.e., streptavidin MicroBeads precipitates out of whole cell homogenates, were applied onto the top of the column matrix and washed 4 times with 100 µL washing buffer to remove non-specifically bound molecules. Elution of target molecules bound to the biotinylated probe was performed by adding 150 µL sample buffer without DTT (non-reducing) directly onto the top of the column matrix. The eluted proteins were heated for 5 min at 95 °C and stored at −20 °C until separated by SDS-PAGE.

### 2.8. Molecular Mass Calculation

The predicted molecular masses of mouse Taar1, the chimeric mTaar1-EGFP, and human TAAR1 were calculated using the SIB Swiss Institute of Bioinformatics ExPASy “Compute pI/Mw tool” (https://web.expasy.org/compute_pi/, accessed on 18 November 2017).

## 3. Results

### 3.1. Constructs and Cell Lines Used for Stable and Transient Expression in Thyrocytes In Vitro

**Figure 1:**
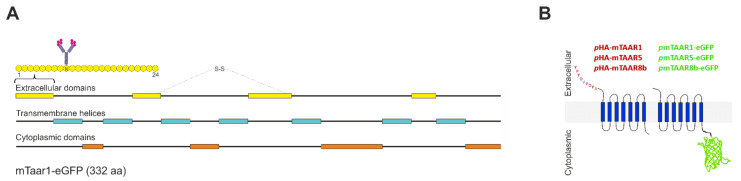
Schemes summarizing the topological structure of mTaar’s. (**A**) A schematic representation of the structural topology of the mouse Taar1, a 332 amino acid long protein, consisting of 7 transmembrane domains (blue), 4 extracellular domains (yellow), and 4 cytoplasmic domains (orange). A putative N-linked glycosylation site on the ninth residue and a disulfide bond connecting the second and third extracellular loops are indicated. The diagram was constructed to annotations in the UniProt entry Q923Y8 (TAAR1_MOUSE). (**B**) A scheme showing the position of the tag in the different Taar chimeric proteins used throughout this study relative to the protein’s orientation in the cell membrane.

The mTaar1 is a 332-amino-acids long, 7-transmembrane GPCR, with extracellular N-terminus and a cytoplasmic C-terminal tail (Figure 1A). In the present study, *mTaar1-EGFP* was studied upon stable expression in human KTC-1 or Nthy-ori 3-1 cells. In addition, N-terminally HA-tagged *mTaar1*, *mTaar5,* or *mTaar8b* were transiently co-expressed in rat FRT cells. A schematic diagram highlighting the position of either tag relative to the protein’s transmembrane orientation is given (Figure 1B).

To test the hypothesis of mTaar1 trafficking in human thyrocytes, stable *mTaar1-EGFP* expression was favored over transient expression because we reasoned that cells require translation of sufficiently high enough protein amounts to facilitate transport to the cell surface, and to enable performing biochemical analyses. This approach was realized by transducing human KTC-1 and Nthy-ori 3-1 thyroid cell lines to express *mTaar1-EGFP*.

Nthy-ori 3-1 is a well-studied human thyroid follicular epithelial cell line that retains functional differentiation, enabling iodide-trapping and thyroglobulin secretion [22]. In contrast, KTC-1 are functionally poorly differentiated papillary thyroid carcinoma cells that, despite not expressing TSH receptors, thyroid peroxidase (TPO) and sodium iodide symporter (NIS), retain high transcript levels of *TG*, *TTF-1* and *paired box gene 8* (*PAX8*) relative to other thyroid cancer cell lines [20,29,30]. Additionally, KTC-1 cells maintain epithelial polarity, supported by the prevalence of tight junction proteins, such as claudin-1, E-cadherin and occludin in the lateral plasma membrane of the cells [14,21]. Both cell lines were therefore regarded suitable to be employed as models for functionally differentiated vs. polarized, structurally differentiated human thyrocytes.

### 3.2. Chimeric mTaar1-eGFP Is Abundant in High Molecular Mass Form in KTC-Z and Nthy-Z Cells, but Primarily Monomeric Chimeras Reach the Surface of KTC-Z Cells

**Figure 2:**
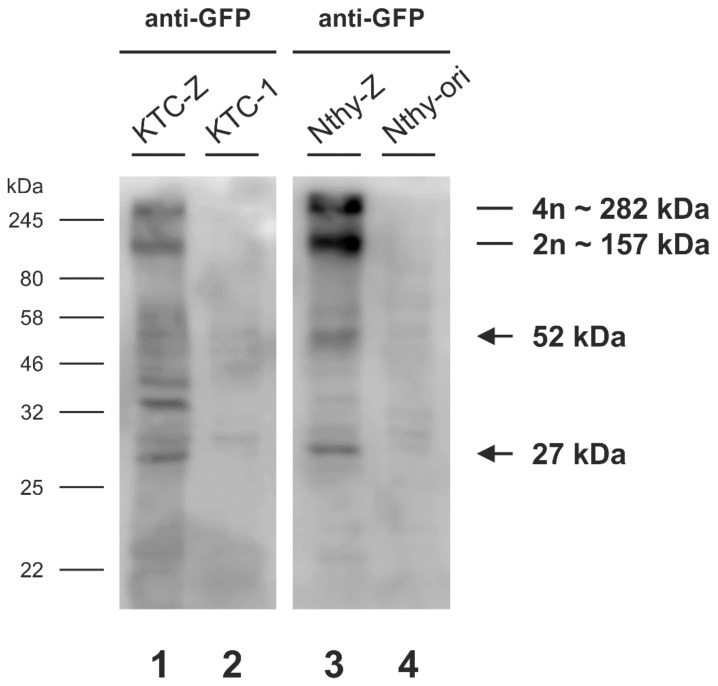
mTaar1-EGFP forms high molecular mass oligomers in stably expressing KTC-Z and Nthy-Z cells. Cell lysates prepared from stably mTaar1-EGFP-expressing KTC-Z and Nthy-Z cells were separated by 12.5% SDS-PAGE along with lysates from non-transduced KTC-1 and Nthy-ori 3-1 cells used as negative controls. Membranes immunolabeled with anti-GFP show two prominent GFP-specific bands at approximately 282 kDa and 157 kDa. An additional band of 27 kDa is observed (arrow), equivalent to the molecular mass of EGFP, and a band at 52 kDa is detected (arrow), which may represent a degradation product of mTaar1-EGFP.

Proteins of whole cell lysates of stably *mTaar1-EGFP*-expressing KTC-Z and Nthy-Z, vs. the non-transduced KTC-1 and Nthy-ori 3-1 controls, respectively, were separated by SDS-PAGE and immunolabeled with GFP-specific antibodies to determine the molecular mass of expressed protein. The predicted molecular mass of mTaar1 equals 37.6 kDa, and mTaar1-EGFP is 65.8 kDa, disregarding potential post-translational modifications like through usage of N-glycosylation sites. Similarly, the predicted molecular mass of human TAAR1 is 39.1 kDa. However, immunolabeling revealed anti-GFP positive bands prominently at an apparent molecular mass of 157 kDa and 282 kDa in KTC-Z and Nthy-Z lanes only (Figure 2). The said molecular masses represent an average of apparent molecular mass values, determined from the exponential equation of retardation factor (Rf) values plotted against the molecular masses of the protein ladder, thereby, potentially representing dimeric and tetrameric forms of mTaar1 (Table 1). This suggests that mTaar1-EGFP exists in SDS-resistant high molecular mass forms, which are not likely resulting from polyubiquitination as no band ladders in 9-kDa spaced pitches are seen.

Additionally, a band at ~27 kDa was seen in *mTaar1-EGFP*-expressing cells only, corresponding to the size of EGFP (Figure 2), indicating cleavage of the EGFP tag upon mTaar1-EGFP degradation. Moreover, a band at 52 kDa was identified in Nthy-Z and KTC-Z cell lysates, only, suggesting degradation products of the chimeric protein (Figure 2).

**Figure 3:**
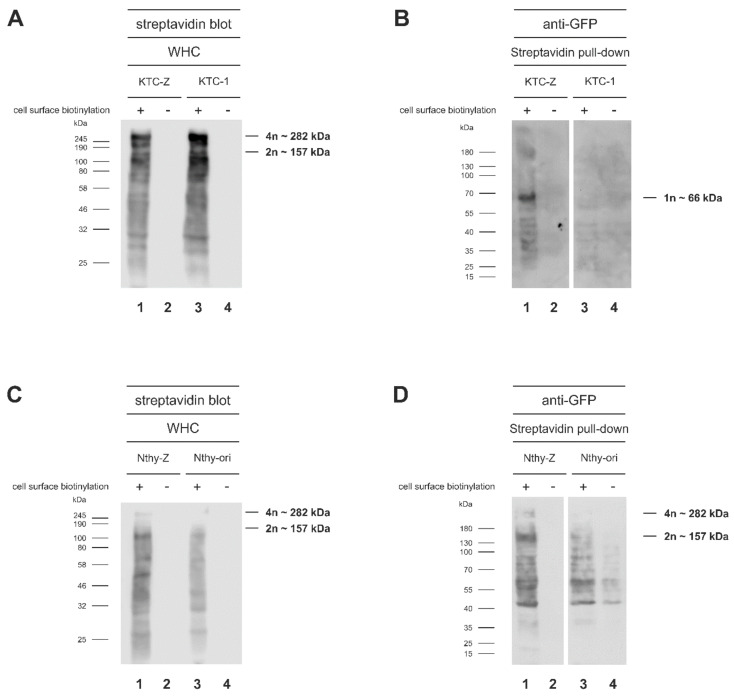
Chimeric mTaar1-EGFP reaches the surface of KTC-Z and Nthy-Z cells. KTC-Z and Nthy-Z cells were subjected to cell surface biotinylation (+) or not (−), cell lysates were separated on 12.5% SDS-PAGE, and blots were labeled with streptavidin conjugated to HRP (**A**,**C**). In addition, streptavidin-coated beads were used to enrich cell surface-biotinylated proteins prior to separation by SDS-PAGE and immunoblotting with anti-GFP antibodies (**B**,**D**). Streptavidin-positive bands equivalent to the GFP-positive bands at 282 kDa and 157 kDa can be detected on the streptavidin blots, particularly in Nthy-Z cell lysates (**C**). A stronger streptavidin signal is seen in KTC-Z cells (**A**, lane 1), suggesting that more mTaar1-EGFP may be reaching the cell surface in KTC-Z than in Nthy-Z cells. To enrich for cell surface-biotinylated proteins, streptavidin pull-down was used with transduced KTC-Z and Nthy-Z cell lysates and their non-transduced controls (**B**,**D**). Preparations from Nthy-Z and Nthy-ori 3-1 cells were not conclusive (**D**). In KTC-Z preparations, however, the anti-GFP antibodies recognized a band of approx. 65.8 kDa in cell surface biotinylated proteins enriched by streptavidin precipitation (**B**, lane 1), indicating cell surface delivery of monomeric mTaar1-EGFP.

Additionally, cell surface proteins of KTC-Z and Nthy-Z cells, as well as of the non-mTaar1-EGFP-expressing KTC-1 and Nthy-ori 3-1 controls, were subjected to biotinylation under endocytosis-blocking conditions at 4 °C. Proteins in lysates from cell surface biotinylated and non-biotinylated control cells were separated by SDS-PAGE and blots were incubated with HRP-conjugated streptavidin to detect biotinylated proteins. The results show that the proportion of biotinylated proteins corresponding in size to the 282 kDa and 157 kDa mTaar1-EGFP tetramer and dimer, respectively, were prevalent in KTC-Z and Nthy-Z cells (Figure 3A,C). However, the abundance of endogenous biotinylated proteins made any further interpretation difficult.

Therefore, an alternative experimental approach was chosen to verify mTaar1-EGFP’s cell surface localization, namely, streptavidin precipitation was performed on cell surface biotinylated lysates of mTaar1-EGFP expressing KTC-Z and Nthy-Z cells, as well as their non-expressing controls (Figure 3B,D). Subsequent anti-GFP immunoblotting was used to identify cell surface-biotinylated forms of mTaar1-EGFP. The results were not fully conclusive for Nthy-Z preparations because many protein bands were identified as streptavidin-precipitated cell surface-biotinylated proteins that were also recognized in the Nthy-ori 3-1 control preparations by the anti-GFP antibodies (Figure 3D, lanes 1, 3, 4). These were in the range of ~40–~130 kDa in Nthy-ori 3-1 controls (Figure 3D, lanes 3, 4) but they did not include the suspected di- or tetrameric forms of mTaar1-EGFP, which were detectable in streptavidin precipitates from Nthy-Z (Figure 3D, lane 1). Specificity of the approach was shown for KTC-Z and KTC-1 preparations because no bands were present for the transduced, non-biotinylated KTC-Z cells (Figure 3B, lane 2) or non-transduced KTC-1 cells (Figure 3B, lanes 3, 4), as expected. The cell surface-biotinylated, transduced KTC-Z cells revealed the 65.8 kDa monomeric form of the mTaar1-EGFP protein in the anti-GFP immunoblots of streptavidin precipitates, while the di- and tetrameric forms were identified as traces only (Figure 3B, lane 1). It is of interest to note that the reverse approach, namely, using anti-GFP antibodies for the precipitation of cell surface biotinylated mTaar1-EGFP forms and their identification on streptavidin blots was not productive with the antibodies used (data not shown).

The results point to the notion that it is mainly monomeric mTaar1-EGFP that reaches the surface of KTC-Z cells in sufficiently high enough amounts to become detectable biochemically (Figure 3B, lane 1). Since biochemistry was not conclusive for cell surface expression of mTaar1-EGFP in transduced Nthy-Z cells, we next used microscopical inspection to visualize mTaar1-EGFP transport.

### 3.3. Transport of mTaar1-eGFP in Transduced, Polarized Thyroid Epithelial Cells Results in Its Targeting to and Localization at Procilia

**Figure 4:**
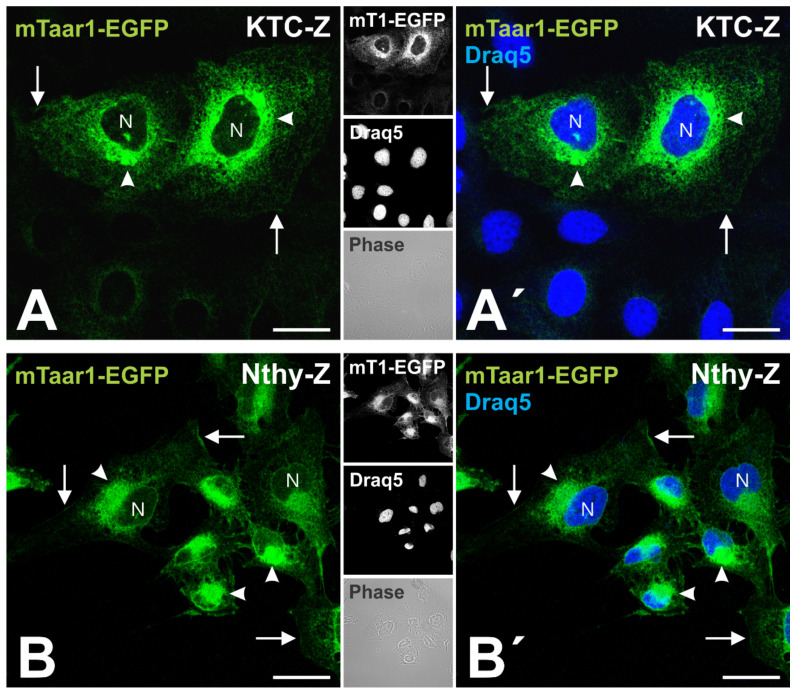
mTaar1-EGFP is predominantly localized in the perinuclear regions of KTC-Z and Nthy-Z cells at 37 °C. mTaar1-EGFP signal can be seen outlining the nuclei (N) and spread out in a reticular pattern, thus, resembling the ER in both KTC-Z (**A**,**A’**) and Nthy-Z (**B**,**B’**) cells. Additionally, mTaar1-EGFP can be seen in the Golgi apparatus (arrowheads). Occasionally, mTaar1-EGFP signal became detectable on the surface of KTC-Z and Nthy-Z cells (arrows). Note that the focal plane of the confocal sections is in the median portion of the cells, which does not allow visualization of cilia. Single channel fluorescence (**A**,**B**) and merged fluorescence micrographs (**A’**,**B’**) for mTaar1-EGFP (green) and Draq5™ as nuclear counter-stain (blue) are shown along with single channel fluorescence and corresponding phase contrast micrographs in the right panels of **A** and **B**, respectively, as indicated. Scale bars represent 20 µm.

When steadily incubated at 37 °C, KTC-Z and Nthy-Z cells exhibit fluorescence in the nuclear envelope in addition to a reticular pattern of mTaar1-EGFP distribution and juxta-nuclear staining of the Golgi apparatus, besides an occasional cell surface localization in sub-confluent cultures (Figure 4). This fluorescence pattern is typical for proteins sorted into the lumen of the endoplasmic reticulum, which is continuous with the lumen of the nuclear envelope, and that are transported along the secretory pathway via the Golgi apparatus [14].

In order to further elucidate mTaar1-EGFP trafficking in the KTC-Z and Nthy-Z cell lines, these were incubated at 18 °C to inhibit anterograde trafficking of proteins along the secretory pathway from the trans-Golgi network (TGN) onwards. Cells were incubated for a minimum of 8 h, and up to 17 h, at 18 °C prior to shifting back to 37 °C to restore the microtubule polymerization-depolymerization dynamics from the perinuclearly located microtubule-organizing center, therefore re-enabling post-TGN vesicle trafficking [31].

**Figure 5:**
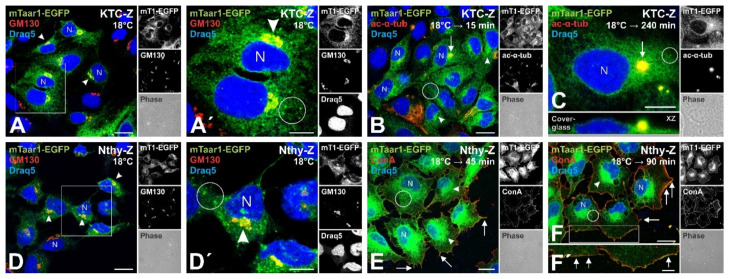
Pulse-chase experiment shows Taar1-EGFP transport along the secretory pathway to procilia of KTC-Z cells. (**A**,**A’**,**D**,**D’**) mTaar1-EGFP is localized in the Golgi (arrowheads) in KTC-Z and Nthy-Z cells directly following incubation at 18 °C. Fixed cells were labeled with antibodies against the cis-Golgi marker GM130 (red). The boxed areas in **A** and **D** are magnified in **A’** and **D’**, respectively. The presence of mTaar1-EGFP (green) in the Golgi of both KTC-Z (**A**,**A’**) and Nthy-Z (**D**,**D’**) cells is evident from its co-localization (yellow) with GM130. mTaar1-EGFP is also seen distributed in a reticular pattern and as puncta (circles), which may represent ER-Golgi intermediate compartments or ER-exit sites. (**B**,**C**) KTC-Z cells were fixed 15 min and 4 h past shift from 18 °C to 37 °C, and immunolabeled with acetylated-α-tubulin. The presence of mTaar1-EGFP (green) on the procilia of KTC-Z cells (arrows) is evident from its co-localization (yellow) with acetylated-α-tubulin (red). Note that procilia are present only above the nuclei and are therefore not detectable in all cells of a given nuclear-near medial focal plane (**B**). In (**C**), a side-view of the same cell is provided as inset (bottom panel), demonstrating the spherical extension representing the procilium (arrow). (**E**,**F**,**F’**) mTaar1-EGFP (green) co-localizes with ConA (red) on the surface of Nthy-Z cells (arrows) at 45 min (**E**) and 1.5 h (**F**) past shift from 18 °C to 37 °C. In (**F’**), a magnified view of the boxed area in (**F**) is shown. (**A**–**F**) Single channel fluorescence micrographs are provided in the right panels, top to bottom: mTaar1-EGFP, GM130 or acetylated-α-tubulin or ConA, as indicated, and corresponding phase contrast. Draq5™ was used as nuclear counter-stain (blue). Scale bars in (**A**,**B**,**D**,**E**,**F**) represent 20 µm, and in (**A’**,**C**,**D’**,**F’**) 10 µm.

Following incubation at 18 °C, mTaar1-EGFP was predominantly observed in the perinuclear region, i.e., in the ER, as indicated by the green mTaar1-EGFP signal outlining the nuclear envelope and surrounding the nuclei in a reticular pattern, as well as in the Golgi apparatus, as evident from co-localization of the mTaar1-EGFP signal with that of the cis-Golgi marker GM130 (Figure 5A,A’,D,D’). Moreover, mTaar1-EGFP was observed as puncta in the cytoplasm of both KTC-Z and Nthy-Z cells (Figure 5A’,D’, circles), which may represent vesicles at the ER-exit sites of the ER-Golgi intermediate compartment (ERGIC) or secretory vesicles in transit to the cell surface.

The ER, Golgi, and vesicular distribution of mTaar1-EGFP was prevalent in both cell lines for the duration of the experiment, i.e., up to 4 h post-shifting the cells back to 37 °C. It should be noted that the chimeric protein persisted in KTC-Z cells upon recovery from the 18 °C transport block, particularly on spherical extensions of the apical cell surface (Figure 5B,C, arrows), consistent with such procilia being resistant to cold temperature conditions (see Discussion). A distinct basolateral cell surface localization of mTaar1-EGFP was observed through co-localization with ConA-stained cell surface constituents in some Nthy-Z cells, especially at 45 min following TGN release onwards (Figure 5E,F, arrows).

It is important to note that procilia localization was assessed by co-localization of mTaar1-EGFP with immuno-stained acetylated α-tubulin which proved a suitable axonemal marker of thyrocyte cell surface protrusions which become well-extended cilia with centrosomal CP110 at their base upon serum-starvation (Supplementary Figure S1).

### 3.4. Incubation with the Putative Ligand 3-Iodothyronamine Does Not Result in Downregulation of mTaar1-EGFP from Procilia or the Cell Surface

**Figure 6:**
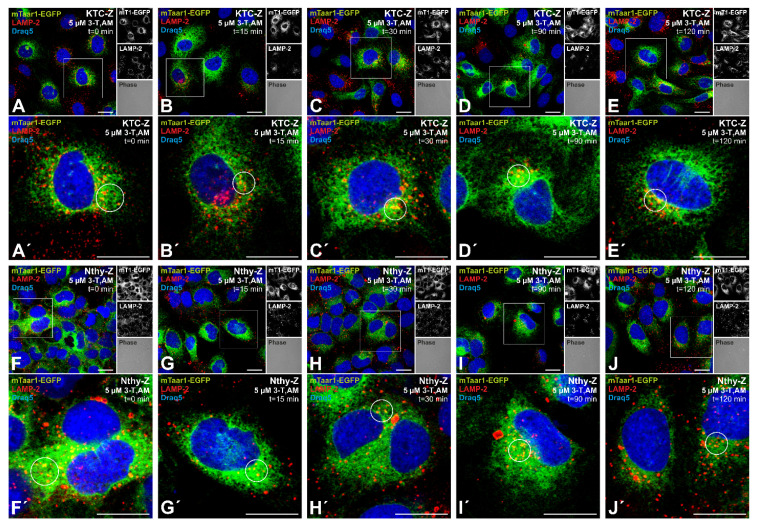
mTaar1-EGFP turnover is constant in steady state and not affected by ligand stimulation. KTC-Z (**A**–**E’**) and Nthy-Z cells (**F**–**J’**) were fixed and immunolabeled with the lysosomal marker LAMP-2 (red) at 0 min to 120 min after stimulation with 5 µM 3-T_1_AM, a potential ligand of TAAR1/Taar1 in the thyroid gland. The boxed areas in **A**–**K** are magnified in **A’**–**J’**, respectively. mTaar1-EGFP (green) partially co-localized with LAMP-2 (red) in endo-lysosomal compartments (yellow) at all time intervals. Circles denote vesicles in which mTaar1-EGFP is seen to co-localize with LAMP-2. Draq5™ was used as nuclear counter-stain. Merged fluorescence (**A**–**J**,**A’**–**J’**) and corresponding single channel fluorescence and phase contrast micrographs are provided in the right panels of (**A**–**J**), respectively, as indicated. Scale bars represent 20 µm.

The morphological transport studies showed that mTaar1-EGFP is trafficked to the cell surface of KTC-Z and Nthy-Z cells, where it was detectable for up to several hours (see above, Figure 4 and Figure 5). The biochemical studies indicated anti-GFP immuno-positive bands that could be representative of degradation products of heterologous mTaar1-EGFP (see above, Figure 2). To understand the fate of mTaar1-EGFP and to test for its possible turn-over, KTC-Z and Nthy-Z cells were immunolabeled with antibodies against lysosomal acidic membrane protein 2 (LAMP-2). Partial co-localization of mTaar1-EGFP with the endo-lysosomal marker LAMP-2 was occasionally observed in constant cultures of KTC-Z cells, while this was less prominent in Nthy-Z cells (Figure 6A,A’,F,F’). These data indicate targeting of the chimeric mTaar1-EGFP protein for lysosomal degradation at steady state, at least in some proportion of the total expressed chimeric protein.

We further reasoned that downregulation of mTaar1-EGFP for subsequent delivery to endo-lysosomes might be triggered by ligand stimulation as typically seen for GPCRs [32]. Therefore, KTC-Z and Nthy-Z cells were incubated with the potential ligand of TAAR1/Taar1, namely, 3-iodothyronamine (3-T_1_AM). The concentration of 5 µM 3-T_1_AM was chosen because it is known to be productive in inducing Taar1 signaling and downstream effects thereof in thyroid epithelial cells in vitro and in situ [33]. Co-localization with LAMP-2 revealed the delivery of mTaar1-EGFP to endo-lysosomes throughout the 2 h past ligand addition (Figure 6). There was no obvious change in mTaar1-EGFP expression, which prevailed in the nuclear envelope and in reticular structures throughout the cytoplasm, while there was also no striking alteration of its endo-lysosomal presence (Figure 6). The data indicated that mTaar1-EGFP turnover remains constant in stably expressing KTC-Z and Nthy-Z cells throughout steady state and irrespective of ligand stimulation or not. This interpretation however assumes that mTaar1-EGFP is functional in transduced human thyrocytes, which must be assessed in future studies.

### 3.5. Serum-Starvation Reveals Transport of mTaar1-EGFP to Cilia of Transduced Human Thyrocytes Arrested at the G_1_/S-Transition

The results described above suggest the notion of mTaar1-EGFP’s transport to primary cilia. However, the structures detected in e.g., KTC-Z cells (see Figure 5C) were not as extended as typically observed in well-differentiated human thyrocytes in situ [2]. In order to recapitulate differentiation states in the G_1_/G_0_-phase of the cell cycle, serum-starvation was used to block cell cycle progression at the G_1_/S transition using a protocol recently established by us for KTC-1 and Nthy-ori 3-1 cells [14]. In addition, the protocol of co-localization of mTaar1-EGFP with marker proteins like acetylated α-tubulin and ARL13B was adapted to sequential fixation of the serum-starved cells with PFA and methanol, respectively, in order to preserve ciliary structures.

**Figure 7:**
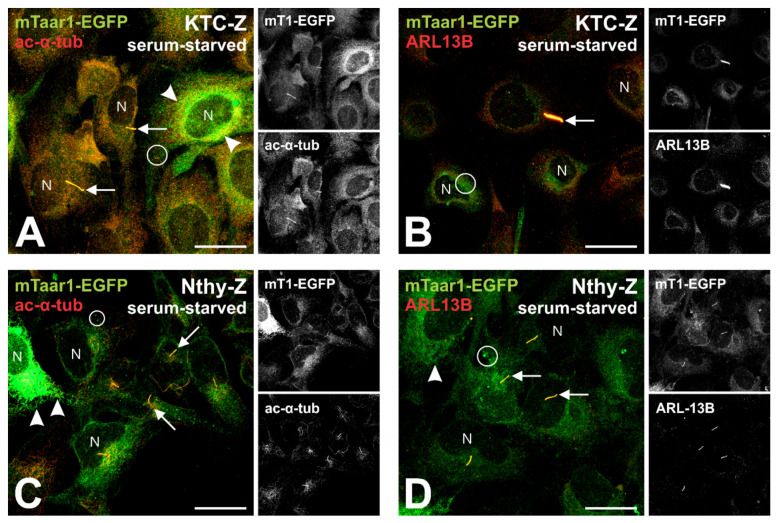
Ciliogenesis and co-localization of mTaar1-EGFP with cilia markers in KTC-Z and Nthy-Z cells. (**A**–**D**) KTC-Z and Nthy-Z cells serum-starved for 48 h (**A**,**B** and **C**,**D**, respectively) were fixed and immunostained with antibodies against the axonemal cilia markers acetylated alpha-tubulin (**A**,**C**, red) or ARL-13B (**B**,**D** red), respectively. The formation of long-extended, acetylated alpha-tubulin- or ARL13B-positive structures (red) emanating from next to or above the nuclei (N) indicates ciliogenesis in the transduced cells arrested at the G_1_/S transition of the cell cycle (arrows). Co-localization with the fluorescence of mTaar1-EGFP (green) indicates the chimeric protein to localize to the compartments of the secretory pathway (arrowheads and circles) and to reach primary cilia of transduced cells (yellow, arrows), indicating targeting and transport of mTaar1-EGFP to cilia of KTC-Z and Nthy-Z cells. Merged (**A**–**D**, left panels) and corresponding single channel fluorescence micrographs of xy-scans (**A**–**D**, right panels, top to bottom: mTaar1-EGFP and acetylated alpha-tubulin in **A** and **C**, or ARL13B in **B** and **D**) are provided as indicated. Arrows point to ciliary extensions, arrowheads to ER or Golgi apparatus, and circles highlight puncta, which may represent ER-Golgi intermediate compartments or ER-exit sites. Scale bars represent 20 µm.

Serum-starvation of transduced KTC-Z and Nthy-Z cells for 48 h resulted in the formation of long, extended structures emanating from above or close to the nuclei, which were immuno-positive for the cilia markers acetylated α-tubulin and ARL13B (Figure 7). Co-localization of the green fluorescent chimeric mTaar1-EGFP protein with the cilia markers acetylated α-tubulin and ARL13B was detected (Figure 7, yellow signals, arrows), besides the presence of the heterologously expressed chimeric protein in the compartments of the secretory pathway, namely, in structures reminiscent of the ER/nuclear envelope, Golgi apparatus and in vesicles (Figure 7, green signals, arrowheads and circles, respectively). We conclude that mTaar1-EGFP is transported to cilia of transduced human thyrocytes in their differentiated states.

### 3.6. Transient Expression of a Related mTaar Protein Results in Trafficking of mTaar1-EGFP to Cilia of FRT Cells

Cell surface transport of mTaar1-EGFP was observed in both the structurally differentiated and polarized KTC-Z, as well as the functionally differentiated but less well-polarized Nthy-Z cells. However, when cultures of transduced cells were maintained in complete medium containing FBS, procilia localization was prevalent in KTC-Z cells only. The fact that mTaar1-EGFP is present mainly in monomeric form at the cell surface of polarized KTC-Z cells, while it reached the surface of Nthy-Z cells possibly as dimers and tetramers (see Figure 3), might argue that the chimeric protein contains both, a cilia targeting and retention signal. Therefore, a co-expression approach was chosen to study the aspect of oligomerization of mTaar1-EGFP with phylogenetically related mTaar’s. The structurally differentiated, well-polarized FRT cells were used for co-expression studies, because transfection of transduced KTC-Z and Nthy-Z cells was not productive. Transduction of FRT cells, on the other hand, was not successful. Therefore, to ask whether homo- and/or hetero-oligomerization between related Taar proteins favors trafficking to the cilia, N-terminally HA-tagged or C-terminally EGFP-tagged *mTaar1*, *mTaar5* or *mTaar8b* (see Figure 1B) were transiently co-expressed in rat FRT cells.

**Figure 8:**
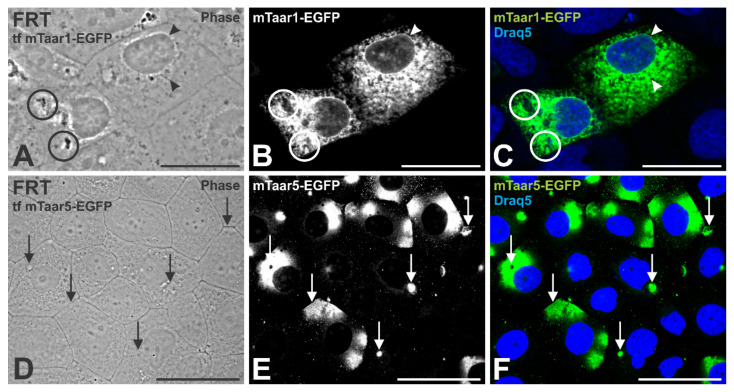
Transient expression of *mTaar1-EGFP* in Fisher rat thyroid (FRT) cells results in endoplasmic reticulum (ER) retention. (**A**–**C**) mTaar1-eGFP has a reticular localization in FRT cells (arrowheads), indicating mTaar1-EGFP (white in **B**, and green in **C**) to be primarily confined to the ER when singly expressed in FRT cells. (**D**–**F**) FRT cells expressing *mTaar5-EGFP* revealed the protein (white in **E**, and green in **F**) to frequently localize at cilia and the lateral borders between neighboring cells (arrows). Phase contrast (**A**,**D**) and corresponding single (**B**,**E**) and merged fluorescence micrographs (**C**,**F**) for mTaar-EGFP (green) and Draq5™ as nuclear counter-stain (blue) are shown. Circles highlight cell debris. Scale bars represents 20 µm in (**A**–**C**) and 50 µm in (**D**–**F**).

When singly and transiently expressed in FRT cells, the mTaar1-EGFP signal appeared in a predominantly reticular and vesicular distribution at steady state, indicating it was retained in the endoplasmic reticulum and did not reach apical cilia (Figure 8A–C). In contrast, mTaar5-EGFP was predominantly localized to cilia and lipid-raft like patches (Figure 8D–F), and it was sorted to the lateral plasma membrane between neighboring FRT cells. Of note, mTaar8b-EGFP was not productively expressed, instead, FRT cell cultures featured cell death upon transient expression of this chimeric protein (not shown).

**Figure 9:**
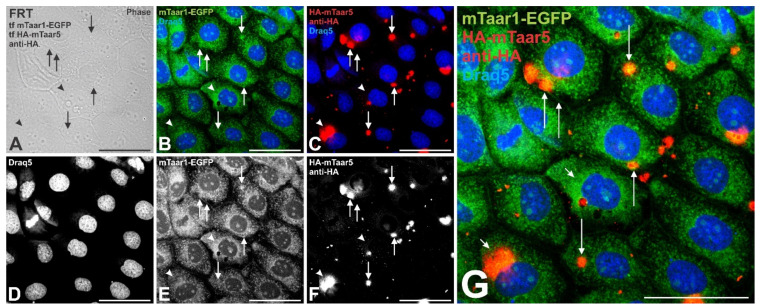
Co-expressing mTaar5 in FRT cells enhances trafficking of mTaar1-EGFP to lipid raft-like microdomains and the ciliary plasma membrane. (**A**–**G**) FRT cells co-expressing *mTaar1-EGFP* (green) and *HA-mTaar5*, labeled with HA-specific antibodies (red in **C**, white in **F**) show co-localization at lipid raft-like microdomains and at cilia (yellow in **G**, arrows) besides a predominant ER-localization of mTaar1-EGFP (green in **B**,**G**, and white in **E**, arrowheads). Merged fluorescence (**B**,**C**,**G**) and corresponding single (**E**,**F**) or phase contrast micrographs (**A**) are provided as indicated, and Draq5™ as nuclear counter-stain in (**B**–**D**). Scale bar represents 50 µm.

Next, we sought to test for Taar hetero-oligomerization and its effect on mTaar1-EGFP trafficking to the cell surface. To this end, an mTaar-EGFP was paired with an HA-tagged mTaar for co-expression studies. Our results show that co-expressing *mTaar1-EGFP* with *HA-mTaar5* results in cilia localization of both (Figure 9B,C,E–G, arrows) in addition to the predominant presence of mTaar1-EGFP in reticular structures reminiscent of the ER (arrowheads). These results indicate HA-mTaar5 expression leading to the partial release of mTaar1-EGFP from ER retention (compare with Figure 8).

The results indicated mTaar5, among the tested mTaar proteins, to be trafficked most efficiently to the apical and basolateral plasma membrane domains of FRT cells. The results suggest that hetero-oligomerization of mTaar1-EGFP with the related HA-mTaar5 promotes trafficking to cilia, while mTaar1-EGFP expression, alone, in the polarized FRT cells was not productive in this regard. Taken together with the results gained with polarized human thyroid cells, the KTC-Z cell line, mTaar1-EGFP transport to the cilia, in particular, is likely in its monomeric form.

## 4. Discussion

TAAR1/Taar1 has been primarily investigated for its neuromodulatory role in the central nervous system, despite being expressed in various human and mouse peripheral tissues [9,10]. As such, it has been assessed as a potential target for pharmacological intervention to treat neurological and psychiatric disorders [34,35,36]; reviewed in [37,38,39]. However, the initially promising therapeutic role of thyronamine-triggered TAAR1 signaling was challenged, among others, by the notion of thyronamines acting as multi-target ligands on several non-GPCRs and GPCRs other than those of the TAAR family [for review, see [12]. Still, in the thyroid, TAAR1/Taar1 might take over a specific role by interacting with the thyronamines that can, in principle, be generated at the lumen-apposed pole of thyrocytes [1]. Therefore, its localization on apical cilia of mouse and rat thyrocytes [1,4] makes it all the more important to understand Taar1 trafficking with the aim to set up human cellular models that will enable studying thyronamine-triggered TAAR1/Taar1 signaling in a thyroid-specific context in future.

Studies revolving around the heterologous expression of Taar1 formerly reported by another group, demonstrated Taar1 to retain an intracellular localization pattern, which led to speculations that Taar1 signals from within intracellular compartments, rather than from the cell surface [10]. Alternatively, this may suggest Taar1 to additionally interact with another protein to facilitate trafficking to the cell surface, a phenomenon known for various other GPCRs [40,41,42]. Indeed, TAAR1 has been reported to form functional dimers with TAAR2 in human leukocytes [43], as well as with human dopamine receptor when co-expressed in HEK 293T cells [44]. Moreover, the majority of studies reporting on TAAR1/Taar1 trafficking and subcellular localization to date entailed N-terminal modifications to the TAAR1 sequence, often to promote its transport to the plasma membrane [10,45,46]. We hereby present a model in which the N-terminus of Taar1 remained intact; however, a covalently linked EGFP tag was introduced at the protein’s C-terminus.

Immunocytochemical analysis revealed mTaar1-EGFP to localize to spherical, procilia structures at the apical plasma membrane of polarized KTC-1 cells, stably *mTaar1-EGFP*-expressing. Surface localization of mTaar1-EGFP was also observed in stably *mTaar1-EGFP*-expressing Nthy-ori 3-1 cells. Pulse-chase experiments showed that prociliary localization of mTaar1-EGFP was maintained even at 18 °C in the polarized KTC-Z cells, as evident from the co-localization with the ciliary marker acetylated-α-tubulin [1,4,47], when cells were inspected shortly after shifting the temperature back to 37 °C to allow post-Golgi transport (see Figure 5). The latter observation suggests that the half-life t_1/2_ of Taar1 at procilia of KTC-Z cells exceeds several hours. In addition, the results suggest that procilia once established in KTC-Z cells are not affected by temperature shifts, which is consistent with the understanding that microtubules of primary cilia are rendered cold-induced disassembly stable by the binding of MAP6 proteins [48]. It is of note, that cilia of rat thyroid epithelial cells are, however, highly susceptible to incubation with cysteine cathepsin inhibitors, causing cilia disappearance and Taar1 re-location to the ER [4]. These data prompted our suggestion of the involvement of ciliary Taar1, co-localized with the thyroglobulin-processing cathepsin proteases, in thyroid auto-regulation (see below) [5].

The observations of this study may at first glance suggest that rodent Taar1 contain a cell surface targeting sequence that is responsible for its transport to reach cilia at the apical thyrocyte pole. However, studies performed on FRT cells transiently transfected with either *mTaar1-EGFP* or *mTaar5-EGFP*, or co-transfected with *mTaar1-EGFP* and *HA-Taar5*, show that, while mTaar1-EGFP was intracellularly retained in transiently expressing FRT cells, mTaar5-EGFP was more readily observed on the cell surface (see Figure 8). However, upon co-expression of both mTaar1-EGFP and HA-mTaar5, partial colocalization was observed at cilia of FRT cells (see Figure 9). Unfortunately, this could not be demonstrated in the human thyrocytes, because transfection and co-transduction of KTC-Z cells were not successful.

From this data, we could speculate that, while intracellular retention was observed when mTaar1-EGFP was transiently expressed in FRT cells, suggesting too low expression levels and homo-oligomerization of Taar1 not being conducive to ciliary targeting, the cilia were reached when *mTaar1-EGFP* was co-expressed with *HA-mTaar5*. Thus, transient co-expression of *mTaar5* constructs in FRT cells in vitro suggest that Taar5 traffics to the surface of well-polarized thyroid epithelial cells more readily than Taar1. The latter may be attributed to the fact that mTaar5 contains the amino acid sequence “FRKALKLLL”, in its C-terminus, which corresponds to the F(X)_6_LL C-terminal motif that was identified to promote GPCR trafficking to the cell surface [49]. This particular motif is absent in the C-terminus of mouse Taar1.

### 4.1. Significance of Taar1 Trafficking to Cilia of Well-Polarized Thyroid Epithelial Cells

The trafficking of mTaar1-EGFP to patches, most likely lipid raft-like microdomains, or ciliary extensions in transfected FRT and in the stably expressing KTC-Z cells as well as in the serum-starved human thyrocytes, which promoted ciliogenesis, supports our previously reported observations that endogenous Taar1 localizes on cilia of FRT cells and on the apical plasma membrane domain of mouse thyroid epithelial cells in situ [1]. This fact is strongly suggestive of Taar1/TAAR1 serving a role in thyroid regulation [for a recent review, see, [5], because the apical plasma membrane domain of thyrocytes faces the thyroid follicle lumen into which the cilia extend and where thyroglobulin, the precursor protein of thyroid hormones, is stored in high concentrations. Therefore, exposing the Taar1 to the extracellular environment opens up the possibility that Taar1 could potentially serve as a sensor to intraluminal molecular alterations. Such changes in the composition of the thyroid follicle lumen are readily achieved upon thyrocyte stimulation with TSH, hence, thyroglobulin degradation may result in the generation of thyronamine precursors, which eventually may be rendered into thyronamines upon cellular uptake and cytosolic conversion before re-export into the lumen, where they can, in principle, act as intra-thyroidally generated Taar1 agonists [1]. This suggestion of an intra-follicular mechanism of Taar1 ligand generation and Taar1 signaling from apically located cilia may contribute to regulating thyroid function in a non-canonical form [1,5,50]. Support of this hypothesis comes from our recent investigations describing the thyroid phenotype of Taar1-deficient mice, which is mild but affects TSH receptor localization in particular [8].

### 4.2. Cilia on Human and Rodent Thyrocytes

Of note, the spherical structures, which we termed procilia, of human thyrocytes KTC-1 and Nthy-ori 3-1 cells kept in complete culture medium are not as well extended as the cilia observed in thyroid follicles in situ [2] because extensive ciliogenesis in vitro requires serum-starvation (see Supplementary Figure S1). This was attempted only in a late phase of this study because we were concerned to not stress the KTC-Z and Nthy-Z cell lines beyond the transduction process. However, our established cell models proved suitable enough to induce ciliogenesis by cell cycle arrest, further supporting our conclusion of having established a valuable in vitro model for future studies. It is further important to note that acetylated alpha-tubulin is an appropriate cilia marker in these cell lines as deduced from comparable staining of peri-nuclear spherical structures in KTC-1 cells with anti-ARL13b and anti-CP110 antibodies (see Supplementary Figure S1), and for its staining of elongated cilia structures in serum-starved cells (see Figure 7).

It is somewhat astonishing that especially KTC-1 cells, which are representatives of human thyroid carcinoma cells, exhibit cilia and maintain mTaar1-eGFP expression at them because in mice, papillary and follicular carcinoma are both correlated with cilia loss [3,7]. Nevertheless, in human thyroid tissue, the disappearance of cilia or their shortening has been associated with hyperactivity of the follicles [2]. It is therefore obvious that the presence of cilia is required to allow trafficking of Taar1 to these appendages of the apical surface of rodent thyrocytes [4], and that a direct connection between the presence of cilia and thyroid cancer is not reproduced by the human cell line KTC-1 (this study).

## 5. Conclusions

This study was conducted by expressing a mouse Taar1 chimera with a C-terminal EGFP tag fused via a short linker peptide. We conclude that KTC-1 and Nthy-ori 3-1 cells stably expressing mTaar1-EGFP provide a suitable model to study Taar1 trafficking and localization in thyrocytes. We report that chimeric mTaar1-EGFP, when expressed in rat and human thyrocytes in vitro, is transported to the cell surface and is preferentially targeted to the primary cilia of polarized thyrocytes, where it exists in monomeric form. We also report that mTaar1-EGFP forms homo-oligomers in stably expressing human KTC-Z and Nthy-Z cells. However, homo-oligomerization was found not to be supportive of ciliary localization of mTaar1-EGFP in our model. We propose these cellular models to be suited for in vivo imaging and signaling studies that are beyond the scope of the present investigation and will be conducted in future. For this to become an even better simulating model, KTC-Z and Nthy-Z cells need to be arrested in the cell cycle at the G_1_/S-transition to achieve full ciliary extensions, which has been demonstrated in this study to be a viable option.

The current study mainly focused on the anterograde trafficking of mTaar1-EGFP from the trans-Golgi network to the cell surface. For future studies, we will rely on these established cellular models to measure mTaar1 turnover rates, i.e., analyze its re-entry by endocytosis and subsequent fates like receptor recycling or endo-lysosomal degradation. Moreover, we intend to perform functional assays to study mTaar1 signaling in vitro, and its implication in regulation of thyroid function. In line with this notion, we have recently discovered that Taar1 is needed to maintain the basolateral localization of the TSH receptor in vivo, suggesting that ciliary Taar1 functionally serves as a co-regulator in the hypothalamic-pituitary-thyroid feedback loop [8].

## Figures and Tables

**Table 1:** Molecular mass measurements from separate anti-green fluorescent protein (anti-GFP) immunoblots of high molecular mass (HMM) bands of mTaar1-EGFP as detected in anti-GFP immunoblots of protein lysates prepared from stably expressing KTC-Z and Nthy-Z cells and separated by 12.5% SDS-PAGE. The mean values, when divided by 65.8 kDa (the predicted molecular mass of the mTaar1-EGFP chimera), yield 2.4 and 2.3 for potential dimeric and 4.4 and 4.2 for potential tetrameric complexes formed in KTC-Z and Nthy-Z cell lines, respectively.

Immunoblot	HMM Band 1	HMM Band 2
KTC-Z Blot 1	182	289.5
KTC-Z Blot 2	135	286.5
KTC-Z Blot 3	164	289
**Means for KTC-Z**	**160.3**	**288.3**
**per 65.8 kDa monomer**	**2.437**	**4.382**
Nthy-Z Blot 1	135	274
Nthy-Z Blot 2	153	307
Nthy-Z Blot 3	167	296
Nthy-Z Blot 4	156	222
**Means for Nthy-Z**	**152.8**	**274.8**
**per 65.8 kDa monomer**	**2.321**	**4.176**
**Average across cell lines**	**157**	**282**
**per 65.8 kDa monomer**	**2.4**	**4.3**

## Data Availability

Not applicable. All data is included and referenced.

## Supplementary Materials

The following are available online at https://www.mdpi.com/article/10.3390/cells10061518/s1, Figure S1: Ciliogenesis and cilia markers in KTC-1 cells.
