# Supplementary material for: Trace Amine-Associated Receptor 1 Trafficking to Cilia of Thyroid Epithelial Cells
Source: Cells. 2021 Jun 16;10(6):1518. doi: 10.3390/cells10061518 (PMC8234161; doi:10.3390/cells10061518)
Supplement: Supplementary file 1 [file cells-10-01518-s001.zip › cells-1241283-supplementary.pdf]

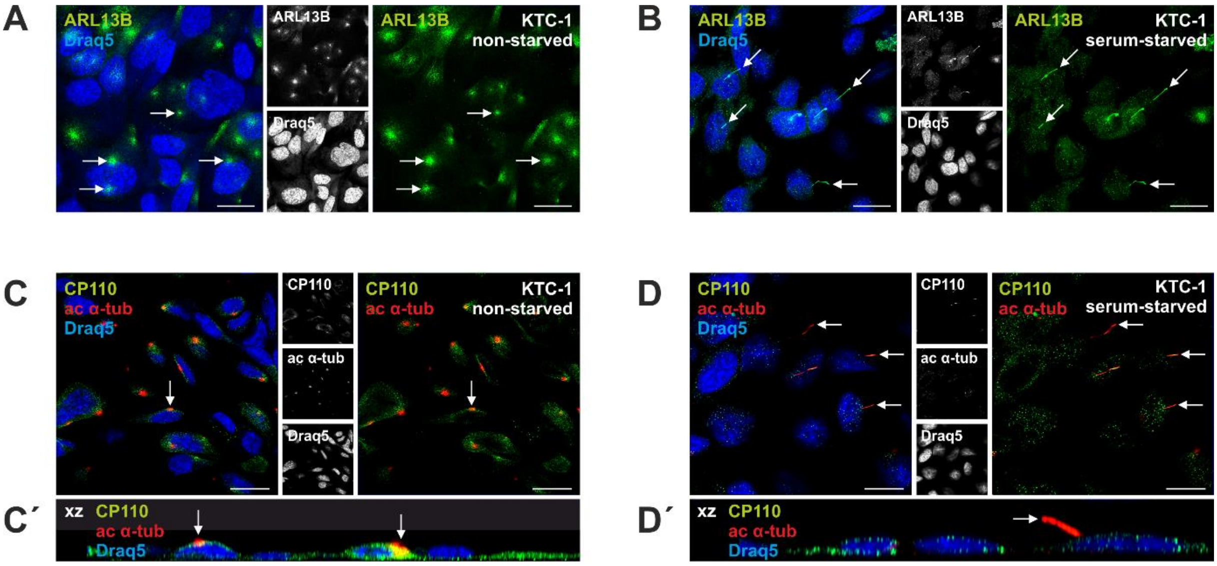

Supplementary Figure 1. Ciliogenesis and cilia markers in KTC-1 cells. (A-D) KTC-1 cells grown in complete FCS-containing culture medium (A,C) and upon 48 h serum starvation (B,D) were fixed and immunostained with antibodies specific for the axonemal cilia marker ARL13B (A,B, green) or acetylated alpha-tubulin (C,D, red), respectively, and the centrosome marker CP110 (C,D, green). (A,B) Circular structures (arrows, green) next to or above the nuclei (blue) denote non-extended cell protrusions of KTC-1 cells in complete culture medium (A). Upon serum starvation, the formation of long-extended, ARL13B-positive structures (arrows) emanating from next to or above the nuclei indicates ciliogenesis in KTC-1 cells arrested at the G1/S transition of the cell cycle (B). (C-D′) Circular structures (arrows) next to or above the nuclei (blue) denote non-extended cell protrusions of KTC-1 cells in complete culture medium in which acetylated alpha-tubulin (red) and CP110 (green) are co-localized at their base (C, yellow as a result of over-lapping red and green signals, arrows), respectively. Immunolabelling with acetylated alpha-tubulin revealed well-extended cilia (red) emanating from CP110-containing regions (green) above the nuclei (blue) of serum-starved KTC-1 cells (D, arrows), particularly obvious in xz-scans depicting side views of the cells (D′). Note that cell protrusions (C′) and extended cilia (D′) are present only above the nuclei and are therefore not detectable in all cells of a given nuclear-near medial focal plane in xy-scans (A-D). Merged (A-D, left and right panels, respectively; C′,D′), and corresponding single channel fluorescence micrographs of xy-scans (A-D, middle panels, top to bottom: ARL13B in A and B, CP110 and acetylated alpha-tubulin in C and D, or Draq5™ used as nuclear counter-stain) are provided as indicated, while the panels C′ and D′ are the corresponding side views along xz from three dimensional optical sectioning in high resolution imaging modes. Scale bars represent 20 μm.
